# FOOL ME ONCE: FRESH PERSPECTIVES ON FINANCIAL VULNERABILITY AND FINANCIAL EXPLOITATION

**DOI:** 10.1093/geroni/igae098.0365

**Published:** 2024-12-31

**Authors:** Julie Miller, Julie Miller

**Affiliations:** Chair; Discussant

## Abstract

Threats associated with fraud and financial exploitation of older adults have grown exponentially. To surface and scale the most meaningful approaches to preventing financial exploitation among older adults, we need innovative approaches that challenge our collective understandings of which factors are at play and what works, why, and how. This symposium will bring together leading scholars to share emerging understandings of mechanisms that fuel financial exploitation, as well as novel approaches with important implications for prevention. The first presentation in the symposium will share new perspectives on cognitive underpinnings of scam susceptibility by exploring individual’s abilities to discern credibility of information and their susceptibility to pseudo-profound statements. The second presentation will describe a randomized control trial with older adult victims of mail fraud designed to evaluate the efficacy of a mailed intervention intended to prevent revictimization. The third and final presentation will highlight an intersectional model of financial exploitation vulnerability that combines interpersonal factors and brain mechanisms, ultimately underscoring the importance of considering multiple factors and different levels of analysis when considering financial exploitation vulnerability in older age. Together, the three presentations will demonstrate the importance of reframing professionals’ and non-professionals’ experiences with − and expectations about − financial vulnerability and financial exploitation.

